# Allergic Fungal Rhinosinusitis in Saudi Arabia: A Review of Recent Literature

**DOI:** 10.7759/cureus.20683

**Published:** 2021-12-25

**Authors:** Abdussalam A AlAhmari

**Affiliations:** 1 Department of Otolaryngology, College of Medicine, Majmaah University, Al-Majmaah, SAU

**Keywords:** kingdom of saudi arabia (ksa), corticosteroid therapy, antifungal drugs, fungal rhino sinusitis, allergic

## Abstract

Allergic fungal rhinosinusitis (AFRS) has been considered an enigma since it was first described four decades ago. Previous research has found that AFRS has multiple definitions and a poorly understood pathogenesis because it overlaps with other conditions and necessitates meticulous work and multiple diagnostic modalities to confirm the diagnosis. However, despite the expansion of medical and surgical treatments, recurrence still occurs. In this review, the recent literature on AFRS cases in Saudi Arabia with relevance to its epidemiology, diagnosis, and management was studied and compared with international data. PubMed, Google Scholar, and Cochrane Library were searched for original research and review articles with local data. There is an evident paucity and contradiction between local studies regarding the epidemiology, diagnostic methods, and management of AFRS. Hence, well-defined randomized controlled trials (RCTs) are needed for the treatment of this chronic recurrent disease.

## Introduction and background

Allergic fungal rhinosinusitis (AFRS) is a non-invasive subtype of chronic sinusitis. It was first described in 1976 as a variant of allergic bronchopulmonary aspergillosis with nasal obstruction [1]. It was first reported as sinusitis in 1981 in a case series of five patients, which was then named "allergic aspergillosis of the paranasal sinuses" [2]. In 1983, after reviewing 119 paranasal sinus specimens, Katzenstein et al. [3] identified it as a new form of chronic sinusitis. It was first described in a pediatric population in 1989 [4]. Robson et al. [5] were the first to introduce the term "allergic fungal sinusitis." The first criteria for diagnosing AFRS were reported by Bent and Kuhn in 1994 [6]. It was composed of five characteristics: the presence of nasal polyposis, radiographic findings, evidence of type I hypersensitivity, eosinophilic mucus without sinus tissue invasion, and aspiration of sinus content yielding positive fungal stain [6]. Despite the prevalence of 5-10% of patients requiring surgery [7], evidence on multiple aspects of this disease remains insufficient.

Environmental factors, susceptible hosts, fungal exposure, and immunity play a role in the pathogenesis of AFRS [8]. Fungal antigens can initiate a cycle of inflammation by inducing type I hypersensitivity. Inflammation leads to mucus hypersecretion and cilia dysfunction. Sinus obstruction and chronic injury will then ensue, causing a niche for fungi to grow and multiply [9].

The aim of this review was to summarize recent local methods for the diagnosis and management of AFRS. In this review, the recent literature on AFRS cases in Saudi Arabia with relevance to its epidemiology, diagnosis, and management was studied and compared with international data.

## Review

Epidemiology

Due to the controversial and varying definitions of AFRS, a clear prevalence of the disease is difficult to obtain. For instance, in Japan, out of 429 patients who underwent endoscopic sinus surgery (ESS), only six patients (1.4%) were diagnosed with AFRS [10]. In contrast, the prevalence is higher in India and the southern parts of the US [11]. It is mostly observed in young men, as they are more likely to be predisposed to risk factors [12]. However, regardless of racial differences in prevalence, bone erosions are common in African Americans [13].

AFRS is commonly reported in areas with warm and humid climates [14]. Ghegan et al. [15] have identified low socioeconomic status and structural anomalies as predisposing factors for AFRS. A higher incidence was observed among people in rural areas because of their work in the agricultural fields in warm weather, exposing their nasal mucosa to injury and fungal colonization [12]. Other predisposing factors include asthma, atopy, and aspirin sensitivity [16].

Our local data showed an AFRS prevalence of 11-14% among patients with chronic rhinosinusitis (CRS) [17-19] and 12% among patients with nasal polyposis [20]. In contrast to worldwide studies, a female preponderance is shown in AFRS cases with a mean male percentage of 38.3%. However, the mean age was 20 years, which is concordant with international data [21-24]. Only two studies examined the geographical variations in prevalence. Al-Dousary [19] reported that most cases were from the Riyadh region. However, the center at which the study was conducted was a tertiary institution in Riyadh, which is a large urban city. The second two areas were Qassim and Jizan, which are agricultural areas in Saudi Arabia [19]. Meanwhile, Al-Bhlal [22] reported that most cases were from the western provinces and that only one was from the southern province. Patients who live in old houses and overcrowded areas are more likely to develop AFRS, as AFRS is most common in patients who live in hot and humid areas such as Jeddah City [25]. Most patients had nasal polyposis diagnosed before or during ESS for CRS [17,19,21-23]. A history of bronchial asthma was reported in 9-36% of patients with AFRS [17,19-22]. Aspirin intolerance was reported as 7.1% by Alghonaim et al. [21] and 1.7% by Al-Dousary [19].

Clinical features

Generally, patients with AFRS present with recalcitrant CRS symptoms that do not respond to typical medical therapy [14]. Most patients present with nasal discharge and headaches [10,12]. The discharge is described as thick, tenacious, greenish-brown mucus with a viscidity similar to that of peanut butter [26]. Almost one-third of adults present with unilateral symptoms [8]. Other less common presenting symptoms are anosmia, facial pain, and postnasal drip [12,27]. Additionally, patients can present with a complicated picture where the lesion extends beyond the sinuses. For example, intraorbital extension through the lamina papyracea may lead to proptosis, visual disturbance, and hypertelorism [14]. A non-invasive extension to the anterior cranial fossa would also present with loss of vision and diplopia by compressing the optic and abducens nerves [28].

Our local data were in concordance with the international data. Most patients had nasal obstruction, nasal discharge, and headache [17,21,22,29]. Bilateral presentation was present in 53-69% of the patients [17,19,21,30]. One of the earliest case reports in Saudi Arabia was for five cases that presented with proptosis without visual field defects [31]. In a study of approximately 40 patients, Al Anazy and Al Dousary [32] suggested a clinical grading system for ophthalmic manifestations. It depends upon the presentation of anatomical changes, disturbed function, the presence of infection, and visual impairment [32]. In another study by Al Dousary [33], he reported 16% of ophthalmic manifestations in patients with AFRS, with proptosis as the predominant presentation. Proptosis was found to be the most common presentation in two separate studies [34,35]. Al-Radadi and Alnoury [36] have reported a case that presented with a visual field defect without changes in visual acuity. Computed tomography (CT) revealed intracranial extension reaching the optic chiasma [36]. Marglani and Shaikh [37] have reported two cases of hemifacial pain that increased during chewing. Imaging showed invasion of the pterygoid plate [37].

Diagnosis

The diagnosis of AFRS is based on a combination of clinical, radiological, microbiological, and histopathological findings. In addition to the Bent and Kuhn criteria mentioned above [6], Kuhn and Swain [38] have suggested the presence of Charcot-Leyden crystals, eosinophilia, asthma, bone erosion, unilateral disease, and fungal culture as minor criteria.

Various authors have developed a scoring system for disease monitoring. Kupferberg and Bent [39] have suggested a post-operative system based on the presence of mucosal edema, allergic mucin, and polyps. Additionally, the Lund-Mackay scoring system for CRS was developed in the mid-1980s and finalized in 1997 [40].

Most recent publications from our region have used the Bent and Kuhn criteria [17,18,21,30,36]. Al-Dousary [19] used the deShazo and Swain criteria and included patients with allergic mucin, detected fungi, without fungal invasion or immunodeficiency [41]. 

Radiological findings

CT has been the preferred diagnostic imaging test for AFRS. Typical findings of the CT scan are sinus opacification, mucocele formation, and skull base erosion [42]. Manning et al. [43] reviewed 10 patients with AFRS, and the CT scans revealed orbital or intracranial extension in most of the patients. However, not all the findings were present in the same patient. For example, Makihara et al. [10] have reported six cases of AFRS without bone erosion.

Salamah et al. [17] have reported 46 AFRS cases with 100% mucosal thickening and 41-58% wall thinning. In the same study [17], wall thinning was directly proportional to disease duration. Al-Dousary [19] reported bony erosions in 35.6% of patients. Al-Swaihb and Al-Dousary [44] revealed 26% bone erosion in 84 patients with AFRS, with a mean age of 20.6 years. Approximately half of the cases had lamina papyracea erosion [44]. Alghonaim et al. [21] have reported 100% involvement of the maxillary sinus with a 3-7% extension outside the sinus. Al-Ghamdi et al. [18] compared CT findings with fungal culture, and its sensitivity was 100%, making it the most sensitive test for diagnosing AFRS. Almomen et al. [45] have presented a rare case in which the ethmoid and sphenoid sinuses were involved without the maxillary sinus. For assessing disease severity, Al Dousary et al. [46] proposed a radiological scoring system that depends on the number and location of bone erosions, with a score ranging from 0 to 72 based on the extent of the erosion.

Magnetic resonance imaging (MRI) can be used to delineate soft tissues further. Generally, hypointense signals are observed in T1 and T2 [42]. The void may be caused by the high protein and low water content of allergic mucin [47]. High peripheral signals in T2 were also observed in some patients [43].

Al-Ghamdi et al. [18] have reported 42% of low-intensity signals in T1 and T2 with post-contrast enhancement. MRI in another case report has helped to show optic chiasm compression [36]. Of all the AFRS cases that underwent MRI, Al-Dousary [19] revealed a 19% extradural intracranial spread of the lesions.

Laboratory findings

Ancillary laboratory investigations can help support the diagnosis. It can be divided into microbiology, histopathology, and serological testing. Evidence of fungal colonization by a stain is considered a major criterion, while that detected by a culture is considered a minor criterion [6,38]. Dematiaceous fungi comprised more than two-thirds of the AFRS cases [48]. Examples of this family include Bipolaris, Curvularia, Alternaria, and Exserohilum. The remaining cases are attributed to the Aspergillus genus [48]. Routine, special, or immunofluorescence staining can help to increase the yield of fungal detection [49]. However, as false positives and false negatives can exist, fungal culture needs to be approached with caution [8]. 

Our local data contradict the findings of the US studies. Fungal hyphae were detected in 64-100% of the histopathological smears [17,19,22]. Al-Dousary [19] has reported a 100% yield of fungal culture with 67% of the Aspergillus genus. The most common species were *Aspergillus flavus* [18,19,50] and *Aspergillus fumigatus* [23,29,31]. Marglani and Shaikh [37] have reported one case of cultured *Aspergillus terreus*. The most common dematiaceous fungi are Bipolaris and Alternaria [19,44]. Al Dousary [19] has reported cases of Penicillium, Saccharomyces, and Epicoccum. Our data are in concordance with reports from the Gulf region and India [51,52].

Histopathology can aid in the diagnosis of AFRS through the presence of eosinophilic mucin and Charcot-Leyden crystals [8]. Demonstration of fungal hyphae can distinguish AFRS from another entity known as eosinophilic mucin rhinosinusitis [16]. However, they are present in almost half of the cases [53]. Another finding in the mucosa is the infiltration of lymphocytes and plasma cells [53]. In addition, AFRS can coexist with other findings, such as granuloma formation. Gupta et al. [54] reported that 15% of patients with AFRS presented with granuloma and raised the theory of granuloma as a progressive form of AFRS rather than a distinct entity.

Specific histopathological findings were found in 30-100% of our local studies [17-19,22,44]. Al Ghamdi et al. [18] compared histopathological findings with fungal culture and identified the presence of allergic mucin as the most specific but least sensitive. Kameswaran et al. [55] have reported four cases presenting with granuloma; however, they did not fulfill the criteria for AFRS. Al Mulhem et al. [56] have reported one case of granuloma in a sample of 15 patients with AFRS. Alarifi et al. [57] have reported a case of AFRS in a patient who previously had chronic granulomatous invasive fungal sinusitis. Although it may be a different pathology, a transformation of previous illness cannot be excluded.

Evidence of type I hypersensitivity can be made by testing serum immunoglobulin E (IgE) levels or by skin prick tests [8]. Serum total or antigen-specific IgE might not be present in all patients with AFRS [38]. Mucin fungal-specific IgE levels were found to be significantly associated with AFRS when compared to serum IgE levels [58]. Although skin testing can be technically difficult, it provides significant evidence for diagnosing AFRS [12].

Peripheral eosinophilia in our local data ranged between 3.4% and 67% [17,19,59]. Of the five children tested for IgE, two had elevated total IgE levels and one had elevated fungal-specific IgE levels [59]. One study compared mean IgE levels between AFRS and CRS and showed a significant elevation in the former group [20]. In a study that compared AFRS, CRS with nasal polyposis (CRSwNP), and healthy controls, patients with AFRS showed a higher total IgE level and specific IgE to *A. fumigatus* [60]. Only one study performed skin testing, and the culture was positive for Aspergillus, Penicillium, and cockroaches [36].

Management

The goal of therapy is to control inflammation and reduce disease burden because this is an inflammatory disease rather than an invasive fungal infection. A comprehensive treatment strategy must include a combination of medical, surgical, and immunotherapy approaches [61].

Medical Therapy

In CRS, systemic corticosteroids are the mainstay of treatment [42]. Corticosteroids, as anti-inflammatory agents, inhibit polyp growth and delay or prevent disease recurrence [42]. However, without surgical treatment, the use of corticosteroids alone is of limited benefit [42]. For example, preoperative prednisolone (1 mg/kg) improved radiological and endoscopic responses in patients with AFRS compared with CRSwNP [62]. Despite the lack of evidence, 39% of ear, nose, and throat (ENT) physicians would use preoperative corticosteroids for patients with AFRS [63]. In a study conducted by Rupa et al. [64], all patients who received postoperative systemic steroids improved in terms of symptoms and endoscopy results after a 12-week follow-up. Gan et al. [65] conducted a systematic review of the literature in 2014 and concluded that postoperative systemic steroids have level B evidence and are recommended in the medical management of AFRS. Some of the side effects of oral steroids include weight gain, poor glucose control, osteoporosis, and cataract formation [65]. Hence, systemic steroids are best used in the perioperative phase and in brief bursts to suppress recurring polyps and manage acute exacerbations of illness [42].

Our data revealed little evidence of preoperative steroid use. Marglani et al. [30] used oral steroids for a shorter period and at a lower dose than the Landsberg et al. [62] protocol. Preoperative steroids were used in 12% of the children, according to Al-Swiahb et al. [59]. Only one study has reported intravenous steroid use, which was most likely due to a presentation with visual loss [36]. Oral postoperative steroid use ranged from 32% to 100% [17,21,44,59].

Topical corticosteroids are also used as a standard treatment for AFRS. These are crucial for the long-term management of AFRS. The benefits include minimal absorption and side effects [61]. They have level A evidence and are recommended for CRS with and without nasal polyposis [66]. Topical corticosteroid monotherapy has not been studied in patients with AFRS. In most AFRS studies, topical steroids were used in conjunction with other modalities, such as oral corticosteroids or surgery [67]. Non-standard, off-label topical steroid treatments, such as high-volume budesonide sinonasal irrigation, offer the theoretical benefit of providing a larger volume and concentration of steroids to the sinonasal mucosa, depending on the method of administration [66]. It has not been studied systematically in AFRS [42], but it is considered an option in some refractory cases [65].

Out of our local data studies that mentioned AFRS treatment, three did not start topical steroids [22,50,55], two started budesonide irrigation [21,30], one specified preoperative use [59], and one continued oral steroids pre-and postoperatively [36]. The percentage of patients who used topical steroids ranged from 57% to 100% [21,23,29,31,44,59]. A study on 17 patients who were started on budesonide irrigation revealed a significant improvement in endoscopic and clinical scores [68]. However, this was an open-label study without a control arm (Table 1).

**Table 1:** Selected local studies showing methods of treatment and outcome. FESS: functional endoscopic sinus surgery.

Study	Number of cases	Medical therapy	Surgical therapy	Outcome
AlQahtani et al. [85]	68 patients	42% received preoperative oral steroids, 57% received postoperative oral steroids, and 23% received budesonide irrigation	44% had conventional FESS* and 56% had extended FESS*	Recurrence rate is 55.8%
Marglani et al. [30]	52 patients	100% received preoperative oral steroids and budesonide irrigation	100% underwent Endoscopic sinus surgery	31.3% had contralateral recurrence and 18.8% had ipsilateral recurrence
Salamah et al. [17]	46 patients	56% received antibiotics, 32% received steroids, and 2.2% received antifungal drops	Type of surgery is not mentioned	30% experienced sinus expansion and 20% experienced wall thinning
Alarifi et al. [84]	40 patients	100% received postoperative oral and intranasal steroids	100% underwent FESS	67.5% had complete bone regeneration
Alghonaim et al. [21]	28 patients	57% received postoperative intranasal steroids, 17% received steroid nasal irrigation, and 43% received oral steroids	39% had unilateral sinus surgery and 60% had bilateral sinus surgery	Recurrence rate is 28.5%

Because patients with AFRS are hypersensitive to fungi, antifungals should theoretically reduce antigenic load and inflammatory responses [65]. It has been suggested that the effectiveness of itraconazole may be related to its anti-inflammatory effects and suppression of steroid metabolism rather than decreased fungal load [61]. A randomized controlled trial (RCT) has found that preoperative itraconazole reduced clinical, radiological, and endoscopic scores in patients with AFRS [69]. Furthermore, according to the RCT by Verma et al. [70], preoperative itraconazole medication produced better outcomes than postoperative therapy. Moreover, antifungals can be used as steroid-sparing drugs in individuals who are contraindicated to taking systemic steroids [71]. Antifungals have adverse effects, and skin, liver enzymes, and electrocardiogram monitoring are required [72]. Thus, oral antifungals should be used for recalcitrant cases [65].

Our local data are in accordance with worldwide recommendations. Older studies have used ketoconazole and amphotericin B for chronic, extensive, or recurrent diseases [22,23,29,31]. Only one study has reported the use of itraconazole [50].

Topical antifungal administration might be a viable option to avoid the toxicity of systemic antifungals [42]. Khalil et al. [73] found that using topical itraconazole following sinus surgery can decrease the recurrence rate of AFRS compared with oral antifungals. However, until more well-designed RCTs suggest the benefit of topical antifungals, they are not recommended for patients with AFRS [65]. Only one study in our local region has reported the use of antifungal drops in one patient, and they did not mention the type or dose [17].

A few therapeutic modalities have been documented in the literature; however, they are still being tested in clinical studies. Leukotriene modifiers are frequently used to lower steroid doses; however, it is uncertain whether they enhance results or minimize the need for revision surgery. There has been only one clinical case report on the impact of leukotriene modulators on AFRS [74]. Biologic drugs are an attractive and promising class of adjuvant therapy for the treatment of CRS, particularly when comorbidities such as asthma are present [72]. Gan et al. [75] treated seven refractory patients with AFRS with omalizumab, a drug that binds specifically to IgE, producing a drop in its levels in both serum and tissue. Manuka honey is a black monofloral honey with high phenolic content that is gaining popularity owing to its antibacterial properties [76]. According to a study by Thamboo et al. [77], SNOT-22 scores improved after 30 days of Manuka honey consumption. The only local data that mentioned additional adjunctive therapy was by Al Dousary. The patients in that study received cefuroxime and clarithromycin for two weeks postoperatively [78].

Since the course of AFRS is prolonged with multiple repeated surgeries and chronic steroid dependence, immunotherapy has been proposed as a steroid-sparing agent and an alternative to surgery [42]. Only a few case reports and retrospective studies have indicated that immunotherapy as a therapeutic option improves polyp formation, systemic corticosteroid usage, and quality of life [79]. Due to its limited availability and high cost, it can be used as an adjunct option by a trained physician in refractory cases [65].

Surgical Therapy

External radical surgery was performed as the primary surgical treatment. However, it is seldom used, except in a limited number of cases [8]. The current approach is performed by endoscopy with the strategy of preserving the mucosal tissue [61]. The goal of surgery is to debride fungal mucin and open sinuses to provide access to deliver topical medication [72]. Additionally, the endoscopic approach aids in the diagnosis by visualizing edema and polyps and providing tissue for histological examination. Furthermore, follow-up endoscopy provides documentation of controlled disease and assesses any recurrence. A newer endoscopic staging system by Philpott et al. [80], which depends on the presence of mucin and edema in the bilateral sinus cavity, correlates better with symptoms than the older Kupferberg system [39].

Studies dated before 2000 relied mainly on external approaches, especially the Caldwell-Luc surgery [22,23,29,31,50]. A study of 22 patients with AFRS has reported performing functional ESS in 50%, computer-assisted sinus surgery in 45.5%, and an external approach in 9.1% of the patients [79]. Al Qahtani et al. [81] have proposed intrapolypoidal white particles (IWP) as an endoscopic sign. In a study of 46 patients with CRS, IWP showed a sensitivity of 85% and a specificity of 65% (Table 1) [81].

Follow-up and recurrence

Since AFRS is a chronic and recurrent disease, clinical and endoscopic follow-up is warranted. Recurrence ranged from 10% to 100%, depending on the duration of follow-up [82]. In a study of 17 patients with a 10-year follow-up, the average number of sinus surgeries was two, and almost 33% of the patients had normal mucosa regardless of the type of treatment [83].

Our local data were consistent with the international data. Recurrence ranges from 8% to 58% [18,21-23,29,31,44,78]. Revision surgery has been reported by Al Dousary [33] in 69% and by Alswaihb et al. [59] in 28% of patients with AFRS. In a follow-up study of 40 patients who had bone erosion with AFRS, two-thirds showed signs of bone regeneration on repeated CT scans [84]. Recurrence of the disease in the contralateral sinus was common. Marglani et al. [30] have reported a 25% contralateral recurrence after a mean duration of two years. Upon studying the risk factors for contralateral recurrence, AlQahtani et al. [85] have reported a significant association between preoperative symptoms and signs of contralateral inflammation (odds ratio (OR): 3.49) and postoperative use of budesonide irrigation (OR: 0.11) (Table 1).

## Conclusions

Our region follows the Gulf and the Indian subcontinent in terms of both the prevalence and causes of AFRS. While most studies have focused on rare presentations or methods of treatment, more data on geographical spread and environmental association is still needed. Further local trials on different treatment modalities are also needed. Over the years, surgical intervention has become less invasive and more targeted than before. However, recurrence and reactivation of the disease were occasionally observed.
